# LC–MS/MS-based quantification of tryptophan, kynurenine, and kynurenic acid in human placental, fetal membranes, and umbilical cord samples

**DOI:** 10.1038/s41598-023-39774-3

**Published:** 2023-08-02

**Authors:** Bruno Pedraz-Petrozzi, Marta Marszalek-Grabska, Anna Kozub, Klaudia Szalaj, Alicja Trzpil, Anna Stachniuk, Eva Kathrin Lamadé, Maria Gilles, Michael Deuschle, Waldemar A. Turski, Emilia Fornal

**Affiliations:** 1 Department of Psychiatry and Psychotherapy, Central Institute of Mental Health, Medical Faculty Mannheim, University of Heidelberg, J5, 68159 Mannheim, Germany; 2 Department of Experimental and Clinical Pharmacology, Medical University of Lublin, Jaczewskiego 8b, 20-090 Lublin, Poland; 3 Department of Bioanalytics, Medical University of Lublin, Jaczewskiego 8b, 20-090 Lublin, Poland

**Keywords:** Biomarkers, Preclinical research, Mass spectrometry, Neurotransmitters, Molecular medicine, Inflammation, Intrauterine growth

## Abstract

Tryptophan breakdown metabolites formed along the kynurenine pathway play a significant role in pregnancy and fetal development. To understand their involvement, it is crucial to quantify the levels of tryptophan (TRP), kynurenine (KYN), and kynurenic acid (KYNA) in relevant biological samples such as the placenta, fetal membranes, and umbilical cord. This study used liquid chromatography-tandem mass spectrometry (LC–MS/MS) to determine TRP, KYN, and KYNA levels. The LC–MS/MS method was optimized for high sensitivity and specificity, demonstrating good reproducibility with a precision of < 10% CV and an accuracy of 85–115%. The lower limit of quantification for both TRP and KYN was 0.5 µg/ml, while for KYNA, it was 0.5 ng/mL. The method exhibited linearity within the examined range of concentrations in the homogenate, ranging from 0.5 to 30 µg/ml for TRP and KYN and from 0.5 to 25 ng/ml for KYNA. Using this method, we found significant differences in the concentrations of these substances in investigated maternal–fetal compartments. Placenta samples exhibited higher KYN and lower KYNA concentrations than the umbilical cord and fetal membrane, indicating a potentially important role for kynurenines in late pregnancy. Collectively, this finding may facilitate further research and provide inside into the involvement of the kynurenine pathway of TRP metabolism in fetal development.

## Introduction

L-Tryptophan (TRP) is an essential amino acid mainly metabolized through the kynurenine pathway (KP)^1^. Under normal conditions, TRP is converted by the hepatic tryptophan 2,3-dioxygenase (TDO-2) and the extrahepatic indoleamine 2,3-dioxygenase (IDO)-1 and IDO-2^2,3^. These enzymes catalyze the conversion of TRP into N-formyl-kynurenine, which can be further metabolized into L-kynurenine (KYN) and its downstream metabolites, ultimately leading to the formation of nicotinamide adenine dinucleotide (NAD^+^)^4^.

The KP enables the formation of many metabolites with differing functions, including the modulation of immunity^5,6^ and inflammation^7,8^. Additionally, these metabolites play a critical role in stress physiology, as daily life stressors can affect the production of TRP breakdown metabolites^9^. One of these metabolites is KYN, an agonist of the aryl hydrocarbon receptor (AhR)^10^. This receptor is ubiquitously expressed in human tissues and involves many metabolic functions. However, its activation also plays an essential role in pathological processes, including inflammation and carcinogenesis^11,12^. KYN serves as the precursor of kynurenic acid (KYNA), an endogenous antagonist of N-methyl-D-aspartate (NMDA) and alpha7 nicotinic acetylcholine receptors, which is involved in inflammation^13^ and is widely studied for its role in various disorders of the central nervous system (CNS)^14–16^. Recent studies have shown that KYNA is an agonist of the G-protein coupled receptor 35 (GPR35)^17^ and AhR^10^. KYNA also serves as a biomarker that directly correlates with stressful events^9,18^. For example, animal models have revealed that stress increases KYNA concentration in the organism over time, resulting in a generalized biological KYNA-dependent stress response^18,19^.

Furthermore, the KP is involved in many physiological processes, playing an essential role in pregnancy by regulating the placental vascular and immune tolerance and providing neuroprotection^1,2,20,21^. For instance, the functional role of IDO in the maintenance of pregnancy in the mammalian placenta was demonstrated in vivo, where inhibition of IDO resulted in pregnancy loss in mice^22^. Additionally, TDO, another key enzyme in the main TRP degradation pathway, maintains fetal and maternal immune tolerance^23^.

Regarding pregnancy and placental tissue, KYN can cross the placenta and the fetal blood–brain barrier. A single oral administration of KYN to pregnant mouse dams raised KYN levels in fetal plasma and brain^24^. In animal studies, continuous KYN supplementation to dams caused memory impairments in adult offspring of animals administered KYN during the pre- and postnatal period^24,25^. KYNA has been shown to play an essential role in fetal growth, particularly in the development of the CNS in utero, as demonstrated by Notarangelo and Schwarcz^26^. Since the placenta is a vital organ in fetal development, serving as the primary source of nutrients and oxygen for the developing fetus, there is a growing interest in understanding the peripheral functions of KYNA in placental tissue physiology. Despite its crucial role in peripheral tissues, the regulation of KYNA in the placenta remains poorly understood. TRP is an amino acid required for protein synthesis and a precursor metabolized along the serotonin and KPs. Interestingly, it was shown a few years ago that TRP is an agonist of G-protein coupled receptor (GPR) 139^27^ and GPR142^28^. Data on the role of these receptors are now remarkably scarce. If the presence of these receptors in pregnancy-associated tissues is confirmed, the measurement of TRP levels will gain new relevance. Therefore, this study aims to investigate the levels of TRP, KYN, and KYNA by presenting a validated method for their quantification in human placental, fetal membranes, and umbilical cord samples using liquid chromatography-tandem mass spectrometry (LC–MS/MS). This method provides a reliable and sensitive tool for studying TRP, KYN, and KYNA in placenta samples. It could offer valuable insights into the regulation of the content of these molecules in the placenta and their potential implications for fetal development.

## Results

In this study, placentae from 9 healthy pregnant women aged 22 to 37 (median: 34, IQR: 4) were included, and the gestational weeks ranged from 37 weeks and 0 days to 40 weeks and 6 days (median: 38 weeks and one day; IQR gestational weeks = 1 and IQR gestational days = 4). No medical complications or infections were reported during or after birth.

**Figure 1:**
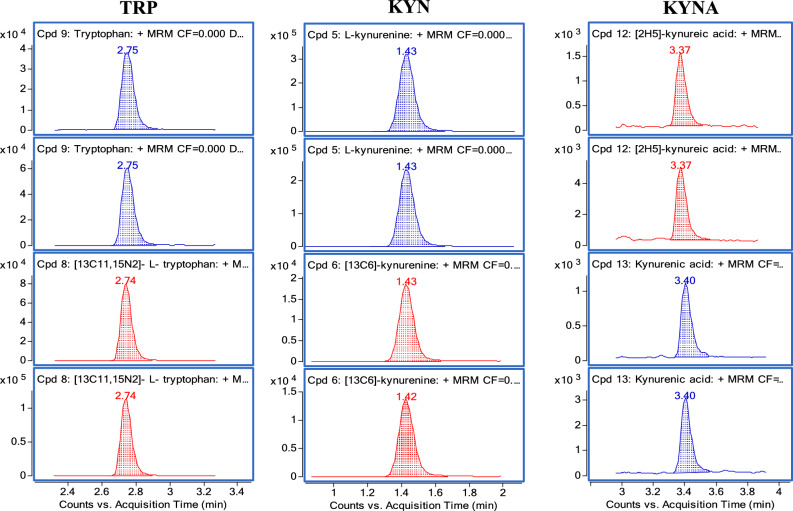
Multiple reaction monitoring of tryptophan (TRP), kynurenine (KYN) and kynurenic acid (KYNA). Analytes are in blue, isotopically labeled standards are in red. Compounds: tryptophan (monitored MRM: 205.1 → 118, 205.1 → 146), [^13^C_11_, ^15^N_2_]-tryptophan (monitored MRM: 218.1 → 127, 218.1 → 156), L-kynurenine (monitored MRM: 209.1 → 94, 209.1 → 146) and [^13^C_6_]-kynurenine (monitored MRM: 215 → 100, 215 → 152), kynurenic acid (monitored MRM: 190 → 116, 190 → 144), [^2^H_5_]-kynurenic acid (monitored MRM: 195.1 → 121, 195.1 → 149).

**Figure 2:**
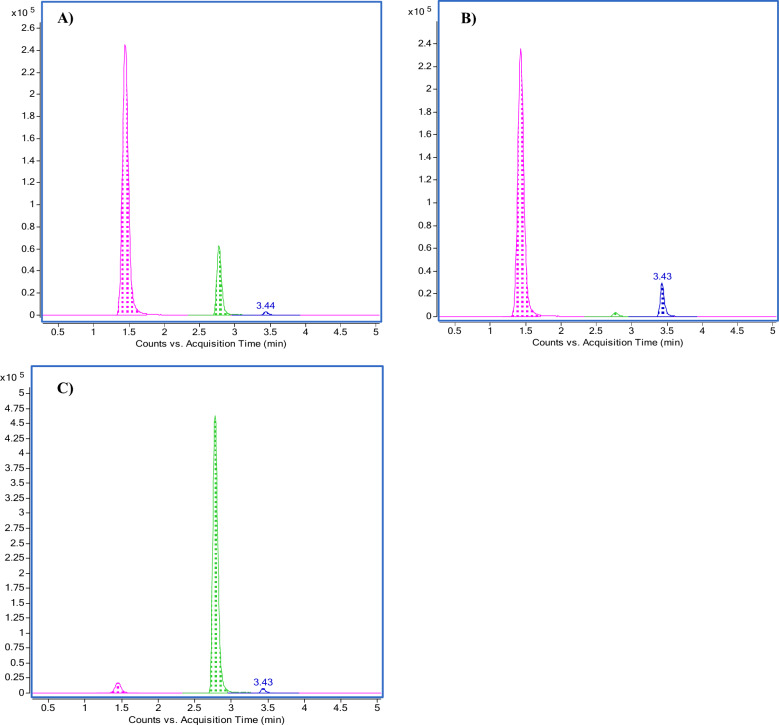
Representative MRM chromatograms for the samples of maternal placenta (**A**), fetal membrane (**B**), and umbilical cord (**C**). In pink: kynurenine (retention time 1.45 min). In green: tryptophan (retention time 2.78 min). In blue: kynurenic acid (retention time 3.43 min).

**Figure 3:**
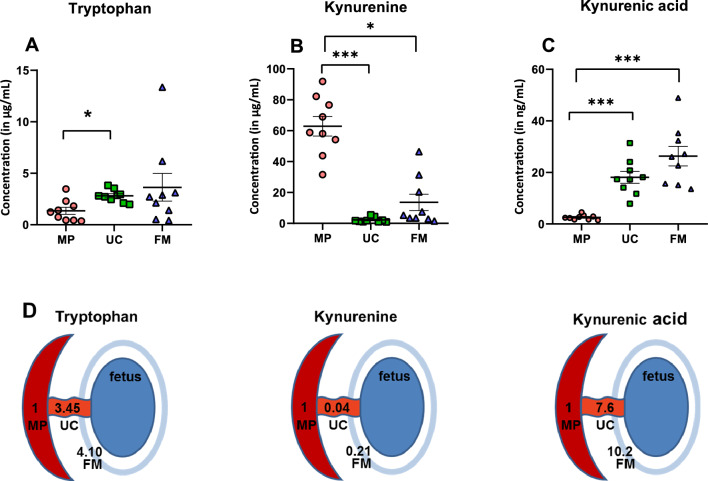
Concentration of tryptophan (**A**), kynurenine (**B**), kynurenic acid (**C**) in maternal placenta (MP), umbilical cord (UC), and fetal membrane (FM) and intercompartmental concentration gradient (**D**). Data are presented as a mean ± SEM, number of subjects = 9 per group (**A**–**C**). The intercompartmental gradient was calculated as the ratio of the investigated substance's umbilical cord or fetal membrane content against its placental content (adopted as 1). *p < 0.05, ***p < 0.001 versus indicated groups. A nonparametric Kruskal–Wallis test indicated a statistical difference in TRP concentration between at least two tissues (*p* = 0.031). Dunn’s multiple comparisons showed lower TRP content in MP versus UC (*p* < 0.05). A nonparametric Kruskal–Wallis test showed a statistical difference in KYN concentration between at least two tissues (*p* < 0.001). Dunn’s multiple comparisons test evidenced lower KYN content in both UC (*p* < 0.001) and FM (*p* < 0.05) in comparison to MP. One-way ANOVA analysis revealed a statistical difference in KYNA concentration between at least two tissues (*p* < 0.001). Tukey’s multiple comparisons tests indicated that the mean KYNA concentration was significantly higher in UC (*p* < 0.001) and FM (*p* < 0.001) in comparison to MP.

LC–MS/MS chromatography was carried out, as detailed in our methods, to measure the concentration of TRP, KYN, and KYNA in the maternal placenta, umbilical cord, and fetal membrane tissue samples. The retention times for TRP, KYN, and KYNA in the three samples were less than 4 min. The chromatograms for the three tissue samples are depicted in Figs. 1 and 2. The analytes exhibited excellent linearity, with regression coefficients (R^2^) greater than 0.99, a precision of less than 10% CV, and an accuracy of 85–115% (Fig. S1). The lowest limit of quantification (LLOQ) was 0.5 µg/mL for TRP and KYN and 0.5 ng/mL for KYNA. The concentrations in the homogenates used for calibration were 0.5–30 µg/mL for both TRP and KYN and 0.5–25 ng/mL for KYNA. Whenever the signal exceeded the range, we diluted the samples before processing or adjusted the injection volume to remain within the range of method linearity. TRP, KYN, and KYNA concentrations were quantified using LC–MS/MS (Figs. 3, S2, S3 and S4). First, we determined the amount of TRP in placental, umbilical cord, and fetal membrane tissues. The concentrations varied between samples, with the highest concentration found in fetal membrane tissues (3.63 ± 1.34 µg/mL), followed by umbilical cord tissue (2.80 ± 0.20 µg/mL) and maternal placenta (1.35 ± 0.35 µg/mL). The difference appeared to be statistically significant between the placenta and umbilical cord. However, no significant differences were found between the fetal membrane, the maternal placenta, and the umbilical cord, probably due to the high variability of intra-group measures (Fig. 3A). The KYN concentrations varied among the samples. The highest concentration was found in maternal placenta tissue samples (62.89 ± 6.35 µg/mL), followed by fetal membrane samples (13.59 ± 5.30 µg/mL) and umbilical cord samples (2.20 ± 0.54 µg/mL). In both umbilical cord and fetal membrane tissues, KYN levels were significantly lower than in the maternal placenta (Fig. 3B). The KYNA concentrations were higher in the fetal membrane (26.30 ± 3.80 ng/mL), followed by umbilical cord tissues (18.08 ± 2.30 ng/mL) and maternal placenta samples (2.58 ± 0.32 ng/mL). In the placenta, KYNA levels were significantly lower than in both umbilical cord and fetal membrane tissue (Fig. 3C). Noteworthy is the considerable value of gradient between the concentrations of the investigated substances in the maternal–fetal compartments studied, which is especially evident for KYN and KYNA (Fig. 3D).

**Figure 4:**
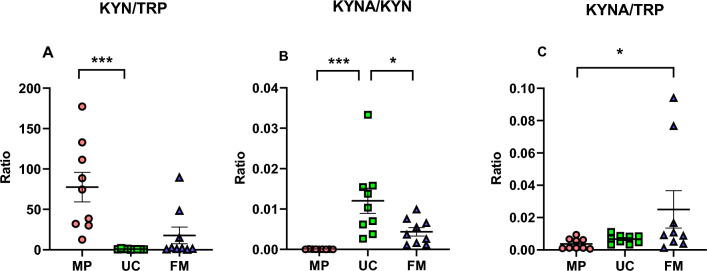
Kynurenine/tryptophan ratio (**A**), kynurenic acid/kynurenine ratio (**B**), and kynurenic acid/tryptophan ratio (**C**) in maternal placenta (MP), umbilical cord (UC) and fetal membrane (FM). Data are presented as a mean ± SEM, a number of subjects = 9 per group. *p < 0.05, ***p < 0.001 versus UC/FM. FM-fetal membrane, KYN-kynurenine, KYNA-kynurenic acid, MP-maternal placenta, TRP-tryptophan, UC-umbilical cord. A nonparametric Kruskal–Wallis test indicated a statistical difference in KYN/TRP ratio between at least two tissues (*p* = 0.001). Dunn’s multiple comparisons test showed a higher KYN/TRP ratio in MP versus UC (*p* < 0.001). Analysis of one-way ANOVA revealed a statistically significant difference in KYNA/KYN ratio between at least two tissues (*p* = 0.001). Tukey’s multiple comparisons test revealed that the KYNA/KYN ratio was significantly higher in UC versus MP (*p* < 0.001) and significantly higher in UC versus FM (*p* < 0.05). A nonparametric Kruskal–Wallis test showed a statistical difference in KYNA/TRP ratio between at least two tissues (*p* = 0.049). Dunn’s multiple comparisons test evidenced a higher KYNA/TRP ratio in FM (*p* < 0.05) than in MP.

Based on these measurements, KYN/TRP and KYNA/KYN ratios were calculated, which reflect the activity of IDO and KAT enzymes, respectively. The KYNA/TRP ratio was also calculated. In the human placenta, we determined a significantly higher KYN/TRP ratio (7755.2 ± 1838.4) in comparison to the umbilical cord (77.5 ± 16.47) and fetal membrane (1777.0 ± 1035.2) (Figs. 4A and S5). KYNA/KYN ratio was significantly higher in the umbilical cord (0.0120 ± 0.0031) than in the placenta (0.000046 ± 0.000009) and fetal membrane (0.0044 ± 0.0011) (Figs. 4B and S6). The KYNA/TRP ratio was higher in the fetal membrane (0.0251 ± 0.0116) than in the maternal placenta (0.0037 ± 0.0011) and umbilical cord (0.0067 ± 0.0009) (Figs. 4C and S7).

## Discussion

To our knowledge, this is the first study to compare TRP, KYN, and KYNA concentrations in pregnant women’s maternal placental, fetal membrane, and umbilical cord tissue samples using LC–MS/MS. Although KYN and its metabolites are known to play a role in fetal development, there is a lack of studies investigating TRP and KP metabolites in these tissues. Most researchers measure the level of substances in the blood, whereas it is crucial to know the content of substances in the tissue, where their potential molecular targets are located.

This report revealed significantly lower KYNA concentrations but higher KYN concentrations in the maternal placenta compared to the other tissues tested. TRP was lower in the placenta than in the umbilical cord. Moreover, the maternal placenta exhibited a significantly higher KYN/TRP ratio than the umbilical cord. On the other hand, the KYNA/KYN ratio was significantly higher in the umbilical cord than in the maternal placenta or fetal membrane. KYNA/TRP ratio was lower in the placenta than in the fetal membrane.

Regarding the technical aspects of the analytical method used to assess TRP, KYN, and KYNA, we achieved an accuracy of 85–115% and a precision of < 10% CV, with an acceptable LLOQ (0.5 µg/mL for TRP and KYN and 0.5 ng/mL for KYNA) and linearity over the studied concentration range. The study of this method meets the criteria for bioanalytical methods, and our results were similar to those of previous studies on the analysis of kynurenines in serum (Hu et al.^29^: < 10% CV; LLOQ: 1 µg/mL for TRP, 100 ng/mL for KYN, and one ng/mL for KYNA; van Zundert et al.^30^: < 13% CV; LLOQ: 0.01 µmol/L for KYN and 0.04 µmol/L for TRP); however there are no studies which assess kynurenines in placental tissue by using LC/MS.

Our study revealed 7- and tenfold lower KYNA concentrations in maternal placenta samples than in the other two analyzed tissue samples, namely the umbilical cord and fetal membrane. The significantly lower concentration of KYNA found in maternal placental samples may have physiological relevance. It has been shown that KYNA does not cross the placental barrier between the mother and the fetus, as evidenced in pregnant mice^31^. However, the concentration of KYNA as high as 1.13 μM in last-trimester human amniotic fluid was reported^32^. This phenomenon may indicate that both compartments are isolated and KYNA production in the fetus occurs independently of the maternal plasma concentration. Indeed, a study conducted on mice tissue slices revealed that the fetal brain might produce 3–4 times more KYNA than the maternal brain and placenta, though less than the maternal and fetal liver^33^. However, it has also been suggested that elevated KYNA levels in the placenta are linked to maternal pathological conditions, such as preeclampsia, and abnormal fetal development, including restricted fetal growth and impaired brain development^26^. Future studies should consider evaluating KYNA concentrations under medical or psychological conditions, such as depression or perinatal psychological stress since KYNA could also play a role in brain development^26^ and its increase is related to stress^26^.

Herein, we show a 5- and 25-fold increase in the concentration of KYN in the maternal placenta compared to the fetal membrane and umbilical cord tissue, respectively. Such marked compartmentalization of KYN points to its local high physiological importance. Mechanistically, this is somehow an unexpected phenomenon because KYN is easily transported across biological barriers. Thus, the underlying cause is presumably local KYN production. Indeed, previous studies have demonstrated that increased IDO levels resulted in an enzymatic activity that leads to an increase in KYN concentration in placental tissues^34^. It has also been shown that KYN production starts in the first trimester, as demonstrated in an ex vivo study with placental cell culture^35^. By the end of pregnancy, the development of the endothelial system in the placenta is responsible for an increase in KYN concentration due to higher IDO enzyme activity^34^. Our KYN/TRP ratio calculation, a substitute of IDO activity, indicated increased activity of this enzyme in the maternal placenta samples, consistent with previous studies^1,34^. Moreover, this enzyme is known to be localized in the syncytiotrophoblast^34,36^ and increases from the 14th week of gestation in the human placenta^1^, reaching higher concentrations by the end of pregnancy^34^. Karahoda and colleagues also reported significant IDO enzyme expression and activity in term placentas^34,37^. Our study is consistent with previous research as we found an increased placental KYN/TRP ratio compared to the umbilical cord samples. In addition, our results are consistent with those of other studies, which highlight the critical role of TDO/IDO in maintaining a pregnancy^22,23^. Our study presented the IDO estimate, calculated using the formula [KYN]/[TRP]. This ratio has been used to express changes in IDO activity in isolated tissues or culture media^38–40^. It is important to note that this indicator does not strictly evaluate enzyme activity in vivo, as relevant physiological conditions are not considered. Instead, the KYN/TRP ratio used in our study is an easy-to-calculate diagnostic index representing the actual content of both substances, which is the sum of the supply, metabolism, and excretion of TRP and KYN.

Other ratios, such as KYNA/KYN ratio and KYNA/TRP ratio, demonstrate dynamic changes in the proportion of KP metabolites in different tissues. Our study found an increased KYNA/KYN ratio, a substitute of kynurenine aminotransferase (KAT) activity, in umbilical cord tissue but not in the maternal placenta or fetal membrane samples. One possible explanation for this finding is the increased amino acid metabolism of the human umbilical cord mesenchymal stem cells, which is essential for its biological function and proliferation^41^. The study of Wang and colleagues reported a high expression of the KAT II isoenzyme in the human umbilical cord mesenchymal stem cells, which catalyzes the conversion of KYN to KYNA^42^. Therefore, the higher KYNA/KYN ratio observed in the umbilical cord tissue sample in our study may be attributed to the mesenchymal stem cells. Further studies are needed to confirm these results and compare KAT activity and its expression between normal-term pregnancies and pathological conditions or dysfunctions such as preeclampsia or perinatal maternal psychological stress. Finally, we find a significant difference in the KYNA/TRP ratio, a marker for the elimination/excretion of kynurenine metabolites indicative of kidney disease^43^, between the placenta and fetal membrane but not umbilical cord samples. The explanation for this phenomenon needs to be substantiated by further research. We speculate that under physiological circumstances, there may be an equilibrium in the placental system regarding the excretion of kynurenine metabolites. Therefore, it would be interesting to compare the excretion of kynurenine metabolites between healthy pregnant participants and those with medical conditions such as preeclampsia or perinatal depression.

Although our results confirm the feasibility of using LC–MS/MS for quantifying TRP and other tryptophan metabolites in the maternal placenta, fetal membrane, and umbilical cord samples, several limitations should be considered. These include the small sample size, the lack of comparison with tissues obtained from patients with medical conditions such as preeclampsia, perinatal depression, or perinatal psychological stress, and the lack of umbilical blood or maternal blood samples for further comparisons. Due to the ethics committee's consent conditions, no person-related data were collected. Therefore, our analysis does not include numerous records, including anesthesia and the use of other drugs during elective cesarean delivery. Technical issues, such as the limit of quantification and analysis throughput, should also be considered limitations. Increasing the assay's sensitivity will be necessary if the concentrations of the metabolites assayed are too low. Automated sample preparation may be considered to increase analysis throughput. However, for small numbers of samples, this throughput is satisfactory. Another limitation of this study was the lack of a comparison with genetic material to analyze the production of enzymes related to TRP breakdown or an immunohistological assessment of receptors targeted by KYNA and other TRP metabolites in our study.

In conclusion, we demonstrated that TRP and its metabolites are measurable reliably in fetoplacental tissues. We have developed a highly sensitive and specific LC–MS/MS method for quantifying TRP, KYN, and KYNA in human placental and umbilical cord samples. The method demonstrated good reproducibility, with < 10% CV precision and 85–115% accuracy. This study underscores the significance of precise TRP, KYN, and KYNA quantification in biological samples and the potential application of this method in future studies investigating the involvement of the KP in fetal development. Additionally, we found a high KYNA/KYN ratio in the umbilical cord, high KYN concentrations, and KYN/TRP ratio but low KYNA concentrations in the maternal placenta using this method in material collected during cesarean sections performed on healthy women.

Future studies could investigate the relationship between the content of kynurenines and various placental and fetal characteristics, such as gestational age, birth weight, and maternal health. Considering the higher content of KYN in the maternal placenta and higher content of KYNA in the fetal compartment and the multiple biological effects exerted by KYN and KYNA, further investigation of their role in the placenta, including synthesis, metabolism, and regulation, would help explain their importance to fetal development. Our method provides a valuable tool for future studies to understand the role of TRP and kynurenines in placental and fetal health and medical or psychological conditions (i.e., perinatal depression or perinatal psychological stress) during pregnancy.

## Materials and methods

### Study design

The following is a methods-based research study with a small sample size of human placenta samples collected anonymously from the Department of Obstetrics at the University Hospital of Mannheim, Germany. This research aimed to analyze the concentrations of TRP, KYN, and KYNA. Additionally, we examined the KYN/TRP ratio (reflecting IDO activity), KYNA/KYN ratio (reflecting KAT activity), and the KYNA/TRP ratio of the human placenta, fetal membranes, and umbilical cord samples. Since the samples were collected anonymously, there was no contact with the participants, and the midwives provided information regarding age and gestation time. The study physician had no contact with the participants. Finally, this study was conducted following the Helsinki Declaration and approved by the Ethics Committee of the Medical Faculty of Mannheim, University of Heidelberg (2022-574).

### Study participants and sample collection

Term placentas (n = 9) were collected after elective cesarean delivery between the 38th and 40th weeks of gestation. All participants who underwent cesarean delivery were medically stable, without any medical conditions, scheduled for the surgical procedure, had previous cesarean delivery experience, and had no medical or emergency indications. Samples of maternal placentae, fetal membranes, and umbilical cords were obtained immediately after birth and dissected. Maternal placentae of patients who underwent emergency procedures and were critically ill or had any medical or obstetric diseases (including fetal death) were excluded from the study.

After the dissection of the placental structure, the biological samples were stored immediately in dry ice for transportation to storage at a research facility at the Central Institute of Mental Health in Mannheim (Germany). The samples were sent in dry ice to the research facility at the Department of Bioanalytics, University of Lublin, Poland, for further analysis.

### Assessment of TRP, KYN, and KYNA

#### Acquirement of chemicals and materials

The analytical stable isotope-labeled standards of TRP [^13^C_11_, ^15^N_2_], KYN [^13^C_6_], and KYNA [^2^H_5_] were acquired from Alsachim (Illkirch-Graffenstaden, France). Acetonitrile and methanol of Optima^®^ LC/MS grade were obtained from Fisher Chemical (Waltham, MA, USA) for use in LC/MS analyses. Formic acid (LC/MS grade) and ethanol (LC/MS grade) were obtained from Merck KGaA (Darmstadt, Germany). Ultra-pure water was acquired from the Millipore Direct-Q3-UV purification system (Merck KGaA, Germany). Titan Syringe Filters RC, 0.2 μm were supplied by Thermo Scientific (Waltham, MA, USA).

#### Standard solution and sample preparation

Analytical stable isotope-labeled standards of TRP [^13^C_11_, ^15^N_2_], KYN [^13^C_6_], and KYNA [^2^H_5_] were prepared as stock solutions at a concentration of 1000 mg/L according to specifications. Internal standard working solutions were prepared in methanol.

For sample preparation, placentas, fetal membranes, and umbilical cords were collected from study subjects after cesarean section and stored at − 80 °C. 0.2 g of each sample was weighed into a 2 mL plastic centrifuge tube and homogenized in ultrapure water (1:3 w/v) using a laboratory ball homogenizer (Bead Mill MAX, VWR, part of Avantor, Radnor PA, United States). Disintegration was performed using 2.8 mm diameter ceramic bead balls at 6 m/s during one cycle of 30 s. After centrifugation at 20,598 × g for 10 min at 4 °C, 25 µL of the working solution of isotopically labeled internal standards: TRP [^13^C_11_, ^15^N_2_], KYN [^13^C_6_], and KYNA [^2^H_5_] were added to 200 µL of the homogenized suspension. The samples were vortexed with 575 µL of chilled methanol/ethanol (1:1 v/v) for 5 min, placed at − 20 °C for 15 min, and then centrifuged for 10 min. The resulting supernatant was transferred to a new centrifuge tube, methanol was added (1:1 w/v), and the samples were vortexed and placed at − 20 °C for 60 min before being centrifuged at 12,100 × g for 20 min at 4 °C. The supernatants were diluted with ultrapure water (1:1 w/v) and filtered through a 0.2 μm syringe filter into autosampler vials.

#### LC–MS/MS analysis

The determination of TRP, KYN, and KYNA was performed using an Agilent Technology liquid chromatograph 1290 Infinity II series coupled to an Agilent Technologies triple quadrupole mass spectrometer 6475 QQQ LC/MS equipped with a Jet Stream ion source. The chromatography was carried out on a Zorbax Extend C18 RRHD column (2.1 × 100 mm, 1.8 µm) with a Zorbax Eclipse Plus C18 UHPLC guard column (2.1 × 5 mm, 1.8 µm) and a gradient of 0.1% formic acid in water and 0.1% formic acid in acetonitrile at a flow rate of 0.5 mL/min. The gradient program was as follows: 0–1 min, 3% B; 1–8 min, 3%-35% B; 8–8.01 min, 35–95% B; 8.01–11 min, 95% B, and then 3 min column conditioning at 3% B. The column and the autosampler were maintained at 40 °C and 10 °C, respectively. The LC/QQQ instrument was operated in positive electrospray ionization (ESI) mode and dynamic multiple reaction monitoring (dMRM) scan type. The MS ion source parameters were set as follows: drying gas temperature 250 °C; drying gas flow 11 L/min; nebulizer pressure 45 psi; sheath gas temperature 200 °C; sheath gas flow 6 L/min and capillary voltage 2750 V. MRM transitions, collision energies (CE) and fragment or voltages for all targeted compounds are presented in Table 1 and Fig. 1. Agilent Mass Hunter Data Acquisition software was used to control the equipment, Mass Hunter Quantitative QQQ Analysis B.10.2, and Qualitative Analysis software B.10.00 were applied for data processing.

**Table 1:** MRM transitions, collision energies, and fragment or voltages for all targeted compounds.

Compound	Monitored MRM (collision energy)	Fragment or voltages
[^13^C_11_, ^15^N_2_]—TRP	218.1 → 127 (33), 218.1 → 156 (21)	88
TRP	205.1 → 118 (33), 205.1 → 146 (21)	88
[^2^H_5_]—KYNA	195.1 → 121 (41), 195.1 → 149 (21)	112
KYNA	190 → 116 (41), 190 → 144 (21)	112
[^13^C_6_]—KYN	215 → 100 (13), 215 → 152 (25)	88
KYN	209.1 → 94 (13), 209.1 → 146 (25)	88

*TRP* tryptophan, *KYNA* kynurenic acid, *KYN* kynurenine.

#### KYN/TRP, KYNA/KYN, and KYNA/TRP ratio

The KYN/TRP ratio representing IDO activity was calculated using the following formula: [KYN]/[TRP]. KYNA/KYN ratio, which represents the enzymatic conversion of KYN to KYNA (considered as KAT activity), was calculated using the following formula: [KYNA]/[KYN]. Finally, we utilized the obtained data to calculate the KYNA/TRP ratio, which serves as a biomarker for evaluating the excretion of metabolites in the KP^43^.

### Statistical analysis

All numeric variables were presented as mean ± standard error of the mean -SEM-). Data that followed a Gaussian distribution were assessed using one-way ANOVA to evaluate the significance of differences between groups. Tukey’s test for multiple comparisons was used as a post hoc analysis if a significant difference was found. Data with a non-Gaussian distribution were evaluated using the Kruskal–Wallis (nonparametric ANOVA) followed by post hoc Dunn’s multiple comparisons test if a significant difference was found. Adjusted *p*-values were calculated for these differences. This study defined statistical significance whenever the two-tail-p-value was less than or equal to 0.05. For the statistical analyses and the descriptive data, we used the R-based software jamovi 2.0.0^44^. Graphs were generated using Prism 8 GraphPad (GraphPad Software Inc., California, United States of America).

### Ethics approval, informed consent and consent to participate

Since the samples were collected anonymously, there was no contact with the participants, and the midwives provided information regarding age and gestation times. The study physician had no contact with the participants. As we exclusively utilized anonymized patient data, individual patient consent was not necessary or possible. The study protocol and all study procedures were reviewed and approved by the ethics committee of the Medical Faculty of Mannheim. . Additionally, this feasibility study was conducted in accordance with the principles outlined in the Helsinki Declaration for non-identifiable samples. Furthermore, it adhered to the definitions for non-identifiable biological materials as set forth by the Committee of Ministers to member states on research on biological materials of human origin of the Council of Europe (958th meeting of the Ministers' Deputies).

Regarding informed consent, we would like to clarify that since the samples were collected anonymously and, consequently, non-identifiable, there was no contact with the participants . The study physician obtained fresh placentas immediately after birth from the procedures and laboratory room of the Department of Obstetrics at the University Hospital of Mannheim. The midwives provided age and gestation time information without names or other identifiable data. The study physician had no contact with the participants. As we only utilized anonymized patient data and did not perform genetic analysis on the placenta samples, individual patient consent was not necessary or possible. This statement and the study protocol received approval from the Ethics Committee of the Medical Faculty of Mannheim, which waived the need for informed consent. The Ethics Committee II of the Ruprecht-Karls-University of Heidelberg (Medical Faculty Mannheim) reviewed the research project (574-22) from an ethical and legal standpoint and did not raise any concerns regarding the implementation. As biomaterial, obtained through residual material, is considered pseudonymous, the Ethics Committee granted the study without informed consent because genetic analyses are not conducted, and personally identifiable data are not utilized. Consequently, contacting the organ donor or accessing medical records for medication or patient-related information was prohibited. Furthermore, the Ethics Committee restricted contact between participants and study team members. Lastly, the material was collected directly from the laboratory of the *Department of Gynecology and Obstetrics* at the University Hospital of Mannheim and was delivered by the hospital's midwives. The committee provided the following instructions, which were strictly adhered to: (1) the project is a feasibility study/methods study using residual material for the establishment of a laboratory method, and (2) since no genetic studies were planned, the biomaterial could be considered pseudonymous at most. Upon completion, the biomaterial was destroyed.

## Supplementary Information

Supplementary Figures.

## Data Availability

The data sets generated and analyzed during the study are not publicly accessible due to the applicable data protection law of the State of Baden-Württemberg, but they are available from the corresponding author on justified request.

## Abbreviations

AhR: Aryl hydrocarbon receptor
ANOVA: Analysis of variance
CV: Coefficient variation
dMRM: Dynamic multiple reaction monitoring
ESI: Electrospray ionization
GPR35: G-protein coupled receptor 35
GPR139: G-protein coupled receptor 127
GPR142: G-protein coupled receptor 142
IDO: Indoleamine 2,3-dioxygenase
KAT: Kynurenine aminotransferase
KP: Kynurenine pathway
KYN: Kynurenine
KYNA: Kynurenic acid
LC–MS/MS: Liquid chromatography-tandem mass spectrometry
LLOQ: Lowest limit of quantification
MD: Mean difference
NAD: Nicotinamide adenine dinucleotide
NMDA: N-methyl-D-aspartate
TDO-2: Tryptophan 2,3-dioxygenase
TRP: Tryptophan

## References

[1] Broekhuizen M, Danser AHJ, Reiss IKM, Merkus D (2021). The function of the kynurenine pathway in the placenta: A novel pharmacotherapeutic target?. Int. J. Environ. Res. Public Health.

[2] Badawy AA-B (2017). Kynurenine pathway of tryptophan metabolism: Regulatory and functional aspects. Int. J. Tryptophan Res. IJTR.

[3] Sedlmayr P, Blaschitz A, Stocker R (2014). The role of placental tryptophan catabolism. Front. Immunol..

[4] Fujigaki H, Yamamoto Y, Saito K (2017). L-Tryptophan-kynurenine pathway enzymes are therapeutic target for neuropsychiatric diseases: Focus on cell type differences. Neuropharmacology.

[5] Schröcksnadel H, Baier-Bitterlich G, Dapunt O, Wachter H, Fuchs D (1996). Decreased plasma tryptophan in pregnancy. Obstet. Gynecol..

[6] Mellor AL, Munn DH (2001). Tryptophan catabolism prevents maternal T cells from activating lethal anti-fetal immune responses. J. Reprod. Immunol..

[7] Cervenka I, Agudelo LZ, Ruas JL (2017). Kynurenines: Tryptophan’s metabolites in exercise, inflammation, and mental health. Science.

[8] Braidy N, Grant R (2017). Kynurenine pathway metabolism and neuroinflammatory disease. Neural Regen. Res..

[9] Chiappelli J (2018). Salivary kynurenic acid response to psychological stress: Inverse relationship to cortical glutamate in schizophrenia. Neuropsychopharmacology.

[10] DiNatale BC (2010). Kynurenic acid is a potent endogenous aryl hydrocarbon receptor ligand that synergistically induces interleukin-6 in the presence of inflammatory signaling. Toxicol. Sci. Off. J. Soc. Toxicol..

[11] Yamamoto J (2004). Characteristic expression of aryl hydrocarbon receptor repressor gene in human tissues: Organ-specific distribution and variable induction patterns in mononuclear cells. Life Sci..

[12] Murray IA, Patterson AD, Perdew GH (2014). Aryl receptor ligands in cancer: Friend and foe. Nat. Rev. Cancer.

[13] Pedraz-Petrozzi B, Elyamany O, Rummel C, Mulert C (2020). Effects of inflammation on the kynurenine pathway in schizophrenia: A systematic review. J. Neuroinflamm..

[14] Ganong AH, Cotman CW (1986). Kynurenic acid and quinolinic acid act at N-methyl-D-aspartate receptors in the rat hippocampus. J. Pharmacol. Exp. Ther..

[15] Kemp JA (1988). 7-Chlorokynurenic acid is a selective antagonist at the glycine modulatory site of the N-methyl-D-aspartate receptor complex. Proc. Natl. Acad. Sci. U. S. A..

[16] Hilmas C (2001). The brain metabolite kynurenic acid inhibits alpha7 nicotinic receptor activity and increases non-alpha7 nicotinic receptor expression: Physiopathological implications. J. Neurosci. Off. J. Soc. Neurosci..

[17] Wang J (2006). Kynurenic acid as a ligand for orphan G protein-coupled receptor GPR35. J. Biol. Chem..

[18] Chiappelli J (2014). Stress-induced increase in kynurenic acid as a potential biomarker for patients with schizophrenia and distress intolerance. JAMA Psychiat..

[19] Pawlak D, Takada Y, Urano T, Takada A (2000). Serotonergic and kynurenic pathways in rats exposed to foot shock. Brain Res. Bull..

[20] Marszalek-Grabska M (2021). Kynurenine emerges from the shadows: Current knowledge on its fate and function. Pharmacol. Ther..

[21] van Zundert SK (2022). The Role of the kynurenine pathway in the (patho) physiology of maternal pregnancy and fetal outcomes: A systematic review. Int. J. Tryptophan Res. IJTR.

[22] Munn DH (1998). Prevention of allogeneic fetal rejection by tryptophan catabolism. Science.

[23] Mellor AL, Munn DH (2004). IDO expression by dendritic cells: Tolerance and tryptophan catabolism. Nat. Rev. Immunol..

[24] Alexander KS (2013). Early developmental elevations of brain kynurenic acid impair cognitive flexibility in adults: Reversal with galantamine. Neuroscience.

[25] Pocivavsek A, Wu H-Q, Elmer GI, Bruno JP, Schwarcz R (2012). Pre- and postnatal exposure to kynurenine causes cognitive deficits in adulthood. Eur. J. Neurosci..

[26] Notarangelo FM, Schwarcz R (2016). Restraint stress during pregnancy rapidly raises kynurenic acid levels in mouse placenta and fetal brain. Dev. Neurosci..

[27] Liu C (2015). GPR139, an orphan receptor highly enriched in the habenula and septum, is activated by the essential amino acids L-tryptophan and L-phenylalanine. Mol. Pharmacol..

[28] Murakoshi M (2017). Discovery and pharmacological effects of a novel GPR142 antagonist. J. Recept. Signal Transduct. Res..

[29] Hu L-J (2017). A simple HPLC–MS/MS method for determination of tryptophan, kynurenine and kynurenic acid in human serum and its potential for monitoring antidepressant therapy. J. Anal. Toxicol..

[30] van Zundert SKM (2023). Simultaneous quantification of tryptophan metabolites by liquid chromatography tandem mass spectrometry during early human pregnancy. Clin. Chem. Lab. Med. CCLM.

[31] Goeden N (2017). Prenatal dynamics of kynurenine pathway metabolism in mice: Focus on kynurenic acid. Dev. Neurosci..

[32] Milart P, Urbańska EM, Turski WA, Paszkowski T, Sikorski R (1999). Intrapartum levels of endogenous glutamate antagonist-kynurenic acid in amniotic fluid, umbilical and maternal blood. Neurosci. Res. Commun..

[33] Notarangelo FM, Beggiato S, Schwarcz R (2019). Assessment of prenatal kynurenine metabolism using tissue slices: Focus on the neosynthesis of kynurenic acid in mice. Dev. Neurosci..

[34] Silvano A (2021). Tryptophan metabolism and immune regulation in the human placenta. J. Reprod. Immunol..

[35] Ligam P, Manuelpillai U, Wallace EM, Walker D (2005). Localisation of indoleamine 2,3-dioxygenase and kynurenine hydroxylase in the human placenta and decidua: implications for role of the kynurenine pathway in pregnancy. Placenta.

[36] Kamimura S, Eguchi K, Yonezawa M, Sekiba K (1991). Localization and developmental change of indoleamine 2,3-dioxygenase activity in the human placenta. Acta Med. Okayama.

[37] Karahoda R (2020). Dynamics of tryptophan metabolic pathways in human placenta and placental-derived cells: Effect of gestation age and trophoblast differentiation. Front. Cell Dev. Biol..

[38] Yasui H, Takai K, Yoshida R, Hayaishi O (1986). Interferon enhances tryptophan metabolism by inducing pulmonary indoleamine 2,3-dioxygenase: Its possible occurrence in cancer patients. Proc. Natl. Acad. Sci. U. S. A..

[39] Ozaki Y, Edelstein MP, Duch DS (1987). The actions of interferon and antiinflammatory agents of induction of indoleamine 2,3-dioxygenase in human peripheral blood monocytes. Biochem. Biophys. Res. Commun..

[40] Takikawa O, Kuroiwa T, Yamazaki F, Kido R (1988). Mechanism of interferon-gamma action. Characterization of indoleamine 2,3-dioxygenase in cultured human cells induced by interferon-gamma and evaluation of the enzyme-mediated tryptophan degradation in its anticellular activity. J. Biol. Chem..

[41] Li H, Dai H, Li J (2023). Immunomodulatory properties of mesenchymal stromal/stem cells: The link with metabolism. J. Adv. Res..

[42] Wang G (2018). Kynurenic acid, an IDO metabolite, controls TSG-6-mediated immunosuppression of human mesenchymal stem cells. Cell Death Differ..

[43] Zhao J (2013). Plasma kynurenic acid/tryptophan ratio: A sensitive and reliable biomarker for the assessment of renal function. Ren. Fail..

[44] Love, J. *et al.* The jamovi project. https://www.jamovi.org/ (2020).

