# Supplementary material for: LC–MS/MS-based quantification of tryptophan, kynurenine, and kynurenic acid in human placental, fetal membranes, and umbilical cord samples
Source: Sci Rep. 2023 Aug 2;13:12554. doi: 10.1038/s41598-023-39774-3 (PMC10397233; doi:10.1038/s41598-023-39774-3)
Supplement: Supplementary file 1 — Supplementary Figures. [file 41598_2023_39774_MOESM1_ESM.docx]

**Supplementary Materials**

**KYNA**

**KYN**

**TRP**

**Figure S1:** Representative examples of calibration curves of the placenta homogenate.

**TRP**

**KYN**

**Figure S2.** Concentration of tryptophan in maternal placenta (A), umbilical cord (B) and fetal membrane (C). Data are presented as a mean ± SEM, number of subjects = 9 per group. FM-fetal membrane, MP-maternal placenta, UC-umbilical cord.

**Figure S3.** Concentration of kynurenine in maternal placenta (A), umbilical cord (B) and fetal membrane (C). Data are presented as a mean ± SEM, number of subjects = 9 per group. FM-fetal membrane, MP-maternal placenta, UC-umbilical cord.

**Figure S4.** Concentration of kynurenic acid in maternal placenta (A), umbilical cord (B) and fetal membrane (C). Data are presented as a mean ± SEM, number of subjects = 9 per group. FM-fetal membrane, MP-maternal placenta, UC-umbilical cord.

**Figure S5.** Kynurenine/tryptophan ratio in maternal placenta (A), umbilical cord (B), and fetal membrane (C). Data are presented as a mean ± SEM, number of subjects = 9~~7-8~~ per group. FM-fetal membrane, KYN-kynurenine, MP-maternal placenta, TRP-tryptophan, UC-umbilical cord.

**Figure S6.** Kynurenic acid/kynurenine ratio in maternal placenta (A), umbilical cord (B) and fetal membrane (C). Data are presented as a mean ± SEM, number of subjects = 9~~7-8~~ per group. FM-fetal membrane, KYN-kynurenine, KYNA-kynurenic acid, MP-maternal placenta, UC-umbilical cord.

**Figure S7.** Kynurenic acid/tryptophan ratio in maternal placenta (A), umbilical cord (B) and fetal membrane (C). Data are presented as a mean ± SEM, number of subjects = 9~~7-8~~ per group. FM-fetal membrane, KYNA-kynurenic acid, MP-maternal placenta, TRP-tryptophan, UC-umbilical cord.
